# Labor may mask a symptom of the rupture of ovarian endometrial cyst: a case report

**DOI:** 10.1002/ccr3.1554

**Published:** 2018-04-26

**Authors:** Tamina Kino, Soichiro Obata, Nana Osanai, Ayasa Hashimoto, Yukiko Okada, Shigeru Aoki, Etsuko Miyagi

**Affiliations:** ^1^ Perinatal Center for Maternity and Neonates Yokohama City University Medical Center Yokohama Japan; ^2^ Gynecology Yokohama City University Medical Center Yokohama Japan; ^3^ Department of Obstetrics and Gynecology Yokohama City University Hospital Yokohama Japan

**Keywords:** Labor pain, ovarian cysts, peritonitis, rupture

## Abstract

As labor may mask a symptom of the rupture of ovarian cyst and delivery is a risk factor of its rupture, the possibility of rupture of ovarian cyst should always be considered during delivery.

## Introduction

In recent years, ovarian endometrial cyst during pregnancy has been on an increasing trend, rising to 0.52% 1. However, ovarian endometrial cysts rarely rupture during pregnancy and there are almost no reports of rupture during labor. Here, we report a case in which, although the ovarian endometrial cyst had ruptured, diagnosis was difficult due to labor pain, and a postpartum ileus resulted.

## Case

**Figure 1:**
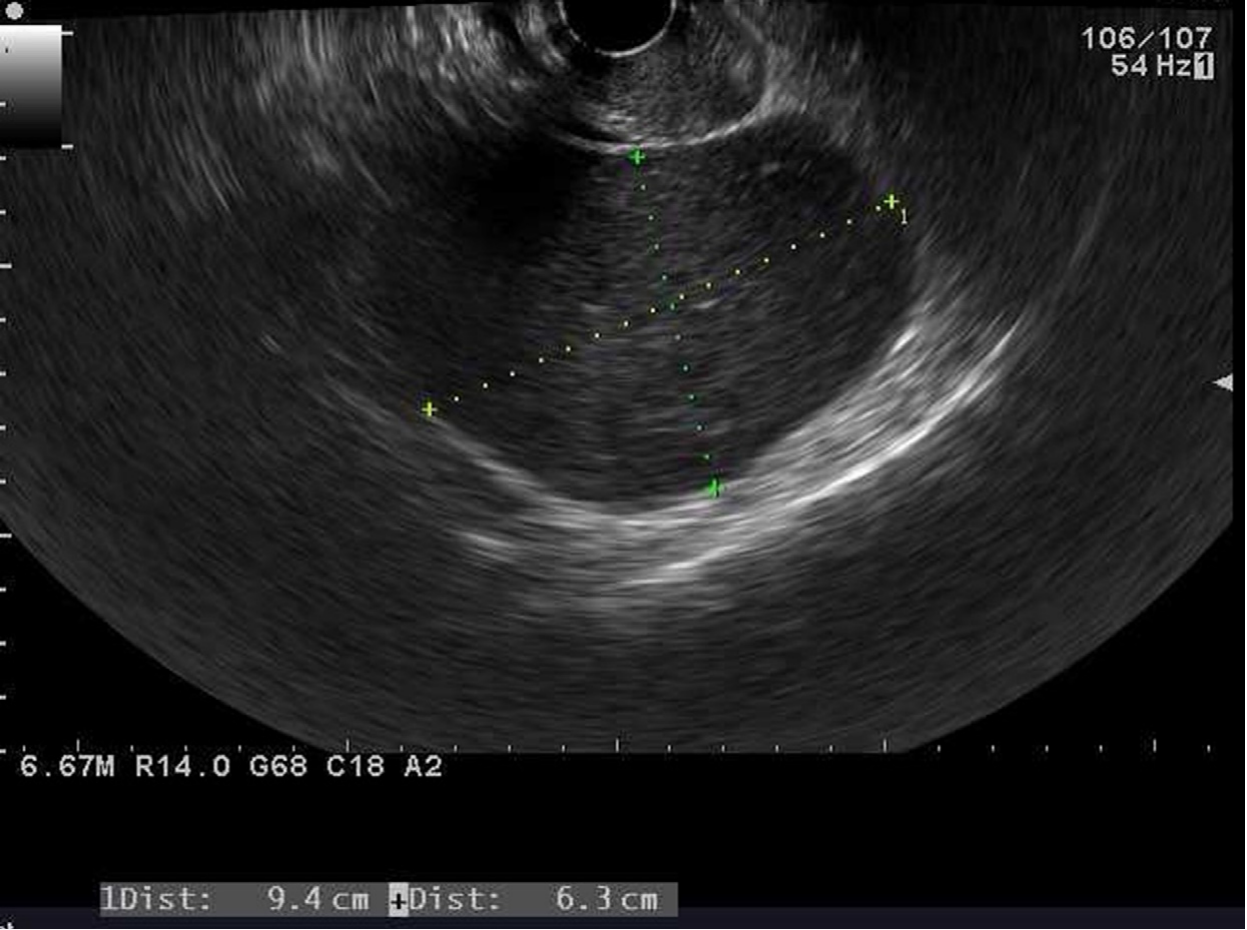
Transvaginal ultrasound image taken at the first visit that revealed a 9.4 × 6.3 cm ovarian endometrial cyst in Douglas' pouch.

Our patient, a 33‐year‐old primigravid, was introduced to our hospital with spontaneous pregnancy complicated by an ovarian cyst. At the initial examination at our hospital, we observed a single 9‐cm cyst in Douglas' pouch that was subsequently diagnosed as an endometrial cyst. As there were no malignant findings, surgery was not performed during the pregnancy (Fig. 1). There were no symptoms and no change in the size of the cyst during the pregnancy. To prevent a post‐term pregnancy, induction of labor by oxytocin was started on the morning of the 41st week and 4th day of pregnancy. At our hospital, we performed epidural analgesia during labor only for patients with complications need analgesia during labor, such as cardiovascular or respiratory disease. Therefore, we do not perform epidural analgesia for this patient.

The oxytocin infusion was discontinued in the evening because no effective labor was achieved despite rupture of the membranes 5 h after labor induction. After the oxytocin infusion was stopped, the uterine contractions continued at 2‐min intervals. The patient had difficulty walking on her own, but this was diagnosed to be due to the labor pain.

**Figure 2:**
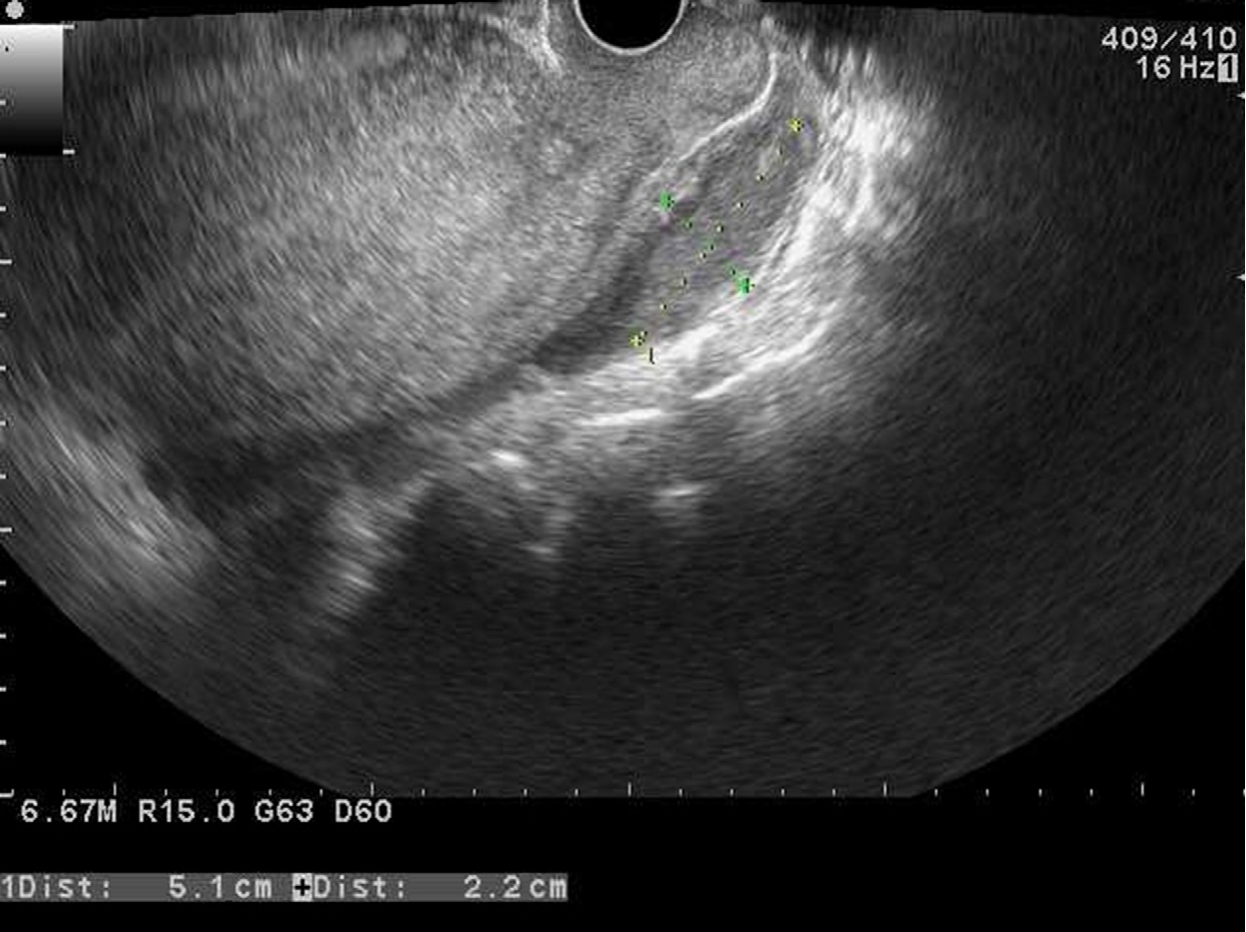
Transvaginal ultrasound image taken 3 days postpartum confirming rupture of the ovarian endometrial cyst in Douglas' pouch.

**Figure 3:**
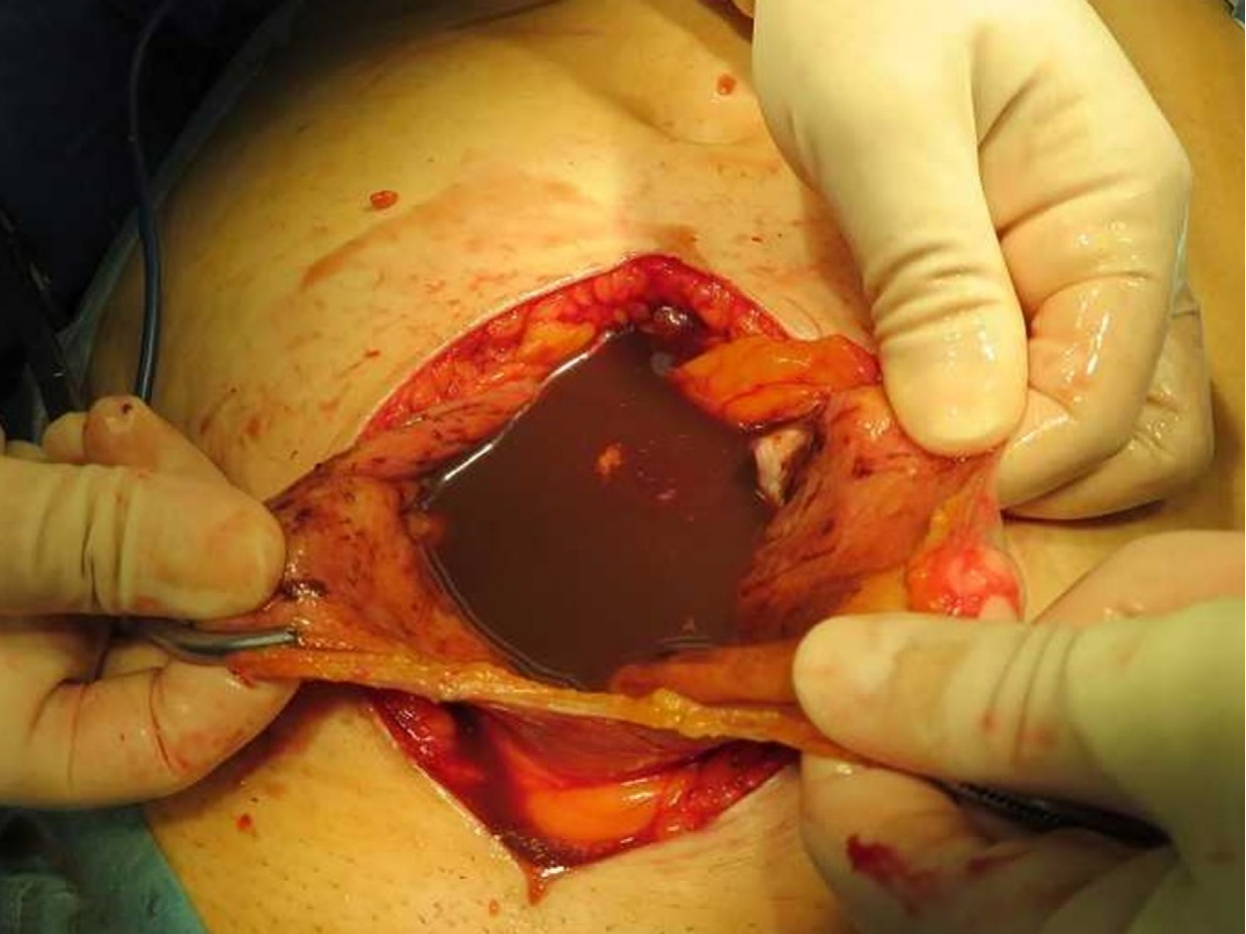
Laparotomy findings revealing pooling of the endometrial cyst contents in the peritoneal cavity.

**Figure 4:**
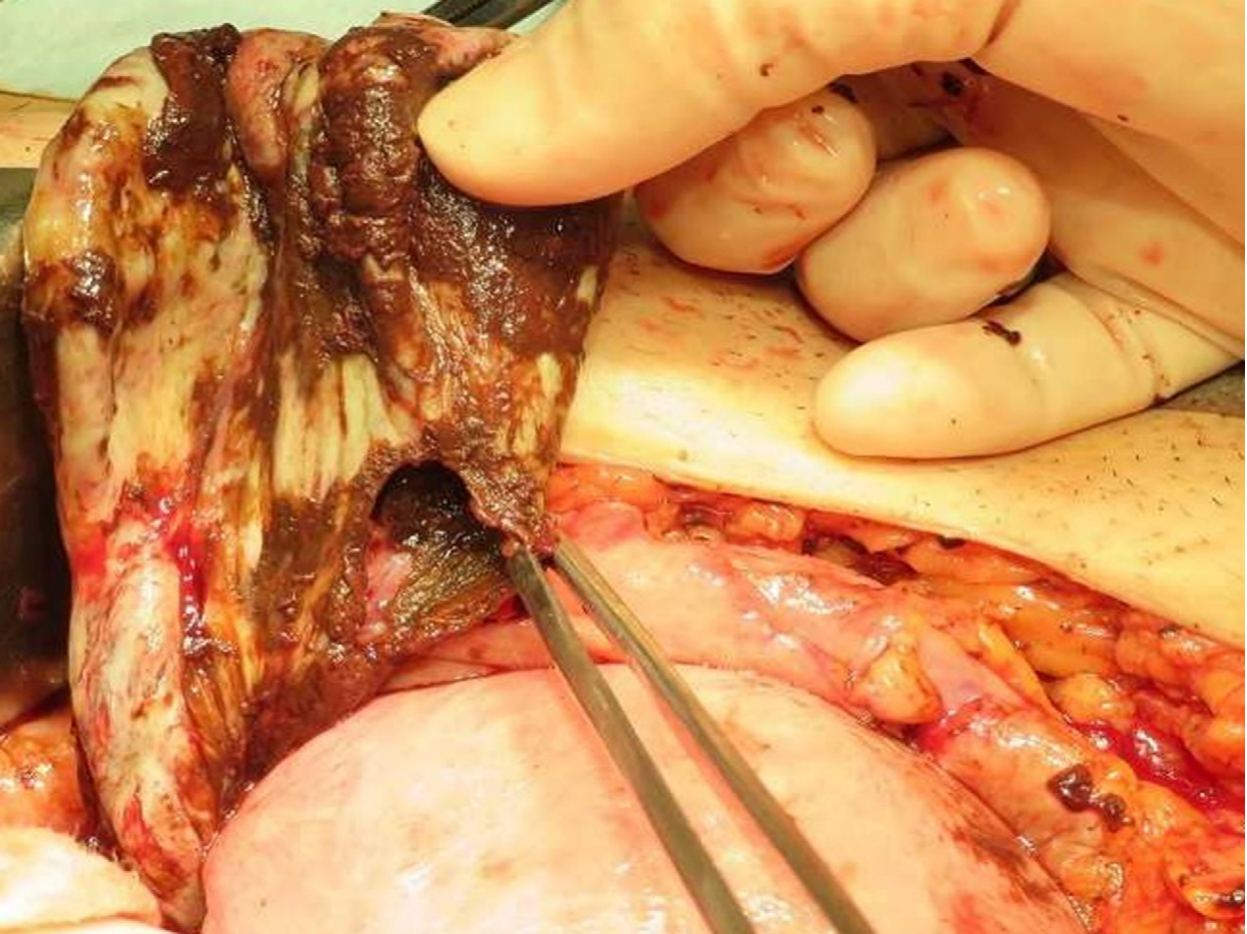
Laparotomy findings confirming rupture of the ovarian endometrial cyst.

**Figure 5:**
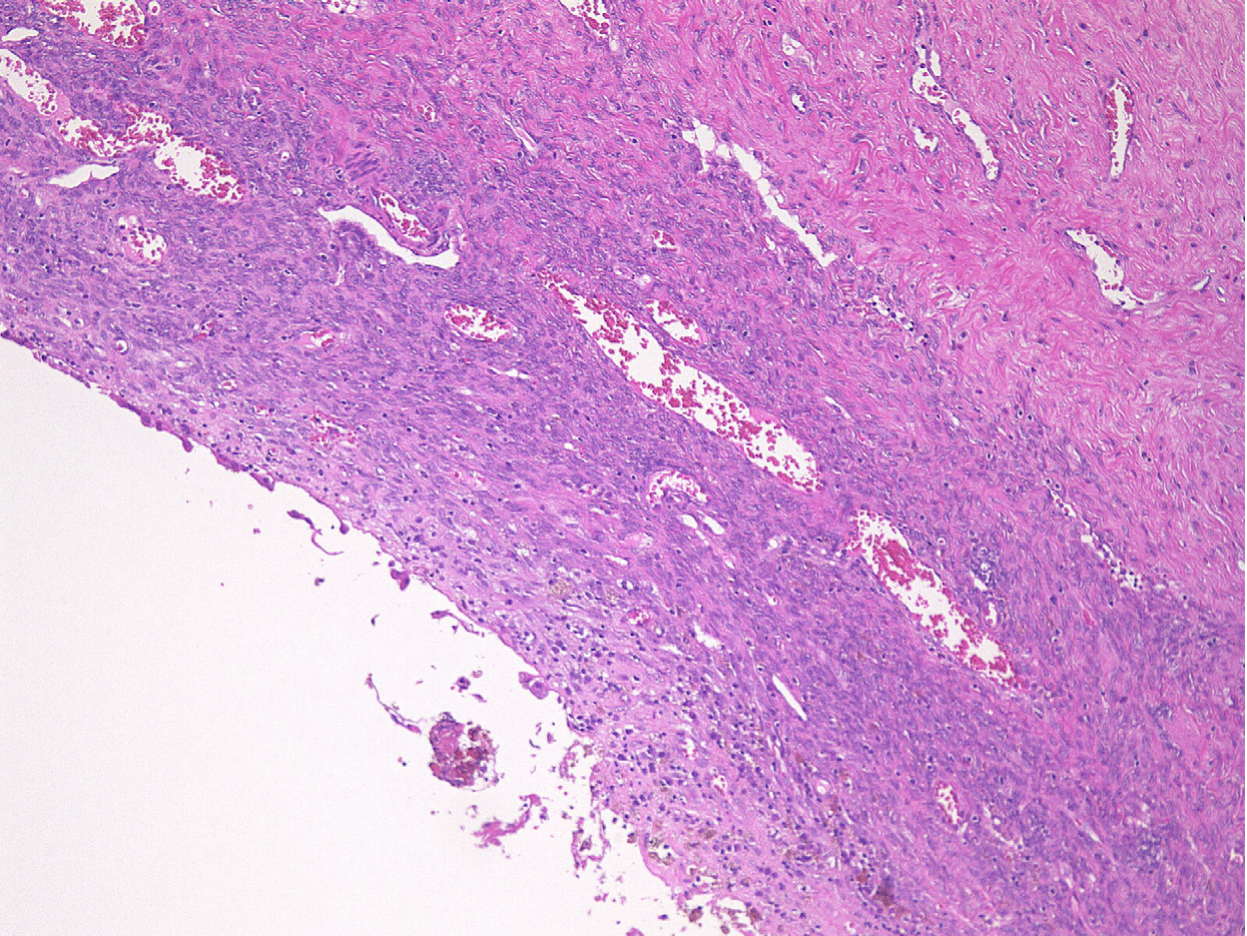
Histologic analysis showed phagocytosis of hemosiderin by macrophage, and diagnosis of endometrial cyst was confirmed (H&E, 100x).

The following morning, delivery progressed to 9 cm of uterine cervix dilation. The patient's body temperature was 36.3°C, but blood sampling revealed a white blood cell count of 26,660/*μ*L and C‐reactive protein level of 14 mg/dL, indicating a high inflammatory response suspected to be due to an intrauterine infection for which cefmetazole sodium was administered at 1 g every 12 h. As the delivery arrested for 2 h after uterine dilation of 9 cm was achieved, the oxytocin infusion was restarted. The baby was delivered the same day. Because the patient complained of lower abdominal pain even after delivery, nonsteroidal anti‐inflammatory drugs were administered. Thereafter, the patient could walk on her own but complained of intermittent nausea and had poor dietary and water intake, so fluid replacement was required from the second day after birth. Three days after birth, the nausea continued, abdominal pain was aggravated, and upper left abdominal tenderness and rebound pain were observed. Ultrasonography revealed ascites retention and reduction in the ovarian cyst (5.1 cm × 2.2 cm) (Fig. 2), and rupture of the ovarian cyst was suspected. Abdominal computed tomography showed collapse of the ovarian cyst and retention of ascites in the upper abdomen corresponding to the ultrasound findings as well as dilation of the intestinal tract. Ileus due to the peritonitis associated with rupture of the endometrial cyst was diagnosed. Laparotomy revealed that the contents of the endometrial cyst had pooled in the peritoneal cavity (Fig. 3), confirming its rupture (Fig. 4). The contents of the cyst were chocolate‐like fluid, and its amount was 125 mL. Hence, a unilateral cystectomy was performed. The postoperative prognosis was good, and the patient was discharged 5 days postoperative. The revised American Fertility Society classification was 30 points preoperatively, and the details were as follows; 20 points for left ovary's deep endometriosis larger than 3 cm, 2 points for its filmy adhesion was 1/3 to 2/3 enclosure, 4 points for its dense 1/3, 4 points for posterior partial cul‐de‐sac obliteration, and 0 point postoperatively. The histologic analysis of the isolated ovarian cyst showed phagocytosis of hemosiderin, and diagnosis of endometrial cyst was confirmed (Fig. 5).

## Discussion

The rupture of ovarian tumors during pregnancy reportedly occurs in 0–5% of the cases 1, 2, 3, 4, while rupture during delivery as in this case is rarely reported. In our patient, the cyst is thought to have ruptured during labor as evidenced by the symptoms of abdominal pain. However, because lower abdominal pain is typical during labor and can mask the symptoms of rupture of ovarian cyst, there was a delay from the onset to diagnosis and a serious course of ileus. As rupture of the ovarian cyst is masked by labor as in this case, it is necessary to monitor patients carefully.

During labor, the fetus descends and places pressure on the tumor. In addition, the uterus contracts after delivery, and when there is adhesion between the tumor and the uterus, the tumor is towed with the contraction. Manual compression and fundal massage in the case of postpartum hemorrhage could also lead to tumor compression, increasing the risk of tumor rupture during or immediately after delivery. Therefore, if there are strong complaints of abdominal pain in cases of pregnancy complicated by ovarian cysts, ultrasonography should be performed to evaluate whether the cyst has ruptured. To our knowledge, there were no previous reports comparing the frequency of rupture of the ovarian cyst between its types. However, adhesion is more likely in endometrial cysts than other types of ovarian cyst 5, 6, and decidualization during pregnancy can affect endometrial cysts resulting in softening to be more likely to rupture 5, 7. Thus, endometrial cyst has the risk of rupture during pregnancy.

It is possible that the primary symptom of rupture of ovarian cyst, sudden lower abdominal pain, may be masked during delivery. The clinical symptom of rupture of endometrial cyst is often lower abdominal pain without signs of infection 8. When rupture occurs during pregnancy, the symptom of lower abdominal pain leads to the diagnosis of rupture 5. In this case, the rupture is thought to have occurred during delivery; because of the labor pains, the abdominal pain upon rupture of ovarian cyst went unnoticed. Furthermore, the pain after delivery was diagnosed to be uterine contraction pain, which further delayed the diagnosis. As a result, it took time to diagnose the rupture, which resulted in ileus with panperitonitis due to the outflow of the cyst's liquid content. Although there is no consensus about surgical indication during pregnancy, it conforms to nonpregnant cases. In endometrial cysts or mature cystic teratoma, those contents may cause chemical peritonitis when the cysts rupture. Thus, surgery may be more recommended in the endometrial cysts or cystic teratoma than other types. In the case of a rupture of ovarian cyst during delivery, the initial symptoms of lower abdominal pain may be overlooked due to labor and the diagnosis may be delayed. Therefore, the possibility of rupture should be considered at all times.

In conclusion, delivery is a risk factor of rupture of ovarian cyst. As lower abdominal pain is a primary symptom of rupture of endometrial cyst, there is a tendency to overlook it as a symptom of labor pain and delay diagnosis as in this case. As a delay in diagnosis may result in severe progression, the possibility of rupture of ovarian cyst should always be considered during delivery.

## Authorship

TK: contributed to the finalization of the manuscript. NO and SO: wrote the first draft of the manuscript. AH and YO: performed the surgery. SA and EM: supervised the case report.

## Conflict of Interest

None declared.
